# Seasonal, spatial, and maternal effects on gut microbiome in wild red squirrels

**DOI:** 10.1186/s40168-017-0382-3

**Published:** 2017-12-21

**Authors:** Tiantian Ren, Stan Boutin, Murray M. Humphries, Ben Dantzer, Jamieson C. Gorrell, David W. Coltman, Andrew G. McAdam, Martin Wu

**Affiliations:** 1 Department of Biology, University of Virginia, Charlottesville, VA USA; 2 Department of Biological Sciences, University of Alberta, Edmonton, Alberta Canada; 3 Department of Natural Resource Sciences, Macdonald Campus, McGill University, Ste-Anne-de-Bellevue, Québec Canada; 4 Department of Psychology and Department of Ecology and Evolutionary Biology, University of Michigan, Ann Arbor, MI USA; 5 Biology Department, Vancouver Island University, Nanaimo, British Columbia Canada; 6 Department of Integrative Biology, University of Guelph, Guelph, Ontario Canada

**Keywords:** Microbial ecology, Biogeography, Dispersal

## Abstract

**Background:**

Our understanding of gut microbiota has been limited primarily to findings from human and laboratory animals, but what shapes the gut microbiota in nature remains largely unknown. To fill this gap, we conducted a comprehensive study of gut microbiota of a well-studied North American red squirrel (*Tamiasciurus hudsonicus*) population. Red squirrels are territorial, solitary, and live in a highly seasonal environment and therefore represent a very attractive system to study factors that drive the temporal and spatial dynamics of gut microbiota.

**Result:**

For the first time, this study revealed significant spatial patterns of gut microbiota within a host population, suggesting limited dispersal could play a role in shaping and maintaining the structure of gut microbial communities. We also found a remarkable seasonal rhythm in red squirrel’s gut microbial composition manifested by a tradeoff between relative abundance of two genera *Oscillospira* and *Corpococcus* and clearly associated with seasonal variation in diet availability. Our results show that in nature, environmental factors exert a much stronger influence on gut microbiota than host-associated factors including age and sex. Despite strong environmental effects, we found clear evidence of individuality and maternal effects, but host genetics did not seem to be a significant driver of the gut microbial communities in red squirrels.

**Conclusion:**

Taken together, the results of this study emphasize the importance of external ecological factors rather than host attributes in driving temporal and spatial patterns of gut microbiota in natural environment.

**Electronic supplementary material:**

The online version of this article (10.1186/s40168-017-0382-3) contains supplementary material, which is available to authorized users.

## Background

Mammalian guts harbor trillions of microbes, which play important roles in diverse aspects of host biology, including nutrition, immune system development, and behavior. Changes in gut microbial composition have been linked to host health and disease [1–4]. Previous studies have shown that host diet, age, sex, genetics, and environmental exposure all drive normal gut microbial variation [4–12]. However, to date, most studies have been focused on human populations or laboratory animals in controlled settings, and much remains to be learned about the ecological forces shaping gut microbial diversity and their relative strengths in nature. Studies of wild animal populations provide important insights into how environment, host biology, and their interactions affect gut microbiota in nature where hosts and microbes have coevolved.

Diet is believed to be a key selective factor in shaping gut microbiota in wild animals. For example, large differences in gut microbial communities have been found among carnivorous, herbivorous, and omnivorous mammals [13, 14]. Wild animals face temporal variation in food availability and often shift their diet accordingly, but little is known about how much this influences gut microbiota. It has been suggested that seasonal variation in gut microbial composition found in wild wood mice [15], ground squirrels [16], and giant pandas [17] are largely driven by the seasonal shifts in diet composition. Nonetheless, these studies either lacked detailed dietary information in the wild or were performed on animals in captivity. Further research is necessary to test if and the extent to which seasonal dietary shifts shape gut microbiota in wild animals. In addition, no study has directly assessed the effect of diet on gut microbiota in wild animals by experimental manipulation of food availability while controlling for the other variables.

Host genetics can also play a role in controlling gut microbial community structure. Accumulating evidence has linked specific host genetic loci to gut microbial variation in humans and mice [18–24]. Although early studies suggested otherwise [6, 25], a recent large-scale study comparing human monozygotic and dizygotic twins revealed significant host genetic effect on gut microbial diversity [9]. In contrast, little is known about the relative contribution of host genetics in shaping gut microbiota in wild animals, as this type of study depends on knowledge of genetic relatedness among many individuals within a wild host population.

While most studies focused on important deterministic factors, little attention has been paid to the role of stochastic processes such as dispersal on structuring gut microbiota. Two recent studies have shown that dispersal limitation could play an important role in shaping gut micriobial communities [12, 26]. With limited dispersal in a homogenous environment, we would predict that nearby hosts would exhibit more similar gut microbe communities than those living farther apart. Variation in gut microbiota over geographical scales has been observed in humans. For instance, family members have a higher degree of gut microbiota similarity than unrelated individuals [25, 27], and distinct gut microbial communities were found in different populations [6, 28, 29]. Likewise, recent studies found biogeographic variation in wild mice populations [15, 30]. However, these patterns do not necessarily indicate dispersal limitation as the only driving force because they can also be attributed to genetic relatedness [30] or shared common environment factors. Furthermore, most studies focused on comparisons between populations separated on large distance scales (average distance > hundreds of kilometers), but few studies investigated the role of dispersal limitation within a host population, where environment is expected to be more homogeneous and less of a concern as a confounding factor.

Recent studies in baboons and chimpanzees have suggested that microbes can disperse within the host population through host social interactions [26, 31, 32]. One particular important mechanism of gut microbiota dispersal is through mother-offspring transmission. Mothers can provide the initial inoculum for the gut microbiota in mammalian newborns. For example, mother koalas produce “fecal paps,” which contain the bacteria necessary to digest gut leaves, and feed them to the young [33]. Accordingly, strong kinship effects were found in several studies [34–36] where the gut microbiota of offspring were more similar to their mother’s than those of unrelated individuals. Using a quantitative genetics approach, it has been shown that maternal effects (non-genetics) can explain as much as 26% of the variation in the gut microbial composition in laboratory mice [23]. However, it is not clear how much of the similarity was due to genetics and how much was due to maternal influences beyond host genetics (i.e., non-genetic maternal effects) in wild animals. Comparative studies in host species with female uniparental care will be useful for separating the maternal effects from the genetic effects.

To determine the relative contribution of seasonal dietary shift, host genetics, maternal effects, and dispersal limitation to the diversity of gut microbiota, we performed a large-scale study on a well-characterized population of wild North American red squirrels (*Tamiasciurus hudsonicus*). As part of the Kluane Red Squirrel Project, every red squirrel in the population has been continuously monitored in each year since 1987, and multiple environmental and host factors were recorded, including age, sex, territory membership, dietary composition, and genetic relatedness [37]. Red squirrels live in a strongly seasonal environment where recurrent seasonal fluctuations in their diet can be carefully tracked using behavioral observations [38]. Both female and male red squirrels defend exclusive territories year round and thus spend most of their time in solitude [39]. As such, red squirrels represent a unique and attractive system to study the effect of dispersal limitation on gut microbial diversity because unlike human and other wild animals studied so far, red squirrels generally remain on their exclusive territory after settlement and have very limited social interactions [40], although they do leave their territory for mating [41] and to pilfer food from neighbors [42]. In addition, females raise young in the absence of any paternal care, making it possible to differentiate host genetics from maternal effects on gut microbiota.

In this study, we tested the hypothesis that red squirrel gut microbiota are strongly influenced by seasonal diet variation. In addition, we tested whether host genetics, maternal effects, or limited dispersal affected gut microbial diversity. We expected not only that genentically related individuals would have more similar gut microbial structures, but also that mothers and offspring were more similar than equally related kin that do not have this close physical contact early in life. Finally, we expected that individuals living in close proximity would have more similar gut microbial composition than those living farther apart.

## Results

### Red squirrel gut microbiota profile

By 16S rRNA gene sequencing, we analyzed the gut microbial communities of North American red squirrels using 905 fecal samples collected from 363 red squirrels. Of these, 622 samples were collected from 230 females and 283 samples were collected from 133 males. Samples varied in both time (collected from February to August between 2008 and 2010) and space (from 6 study grids that were geographically separated from each other by 0.2~7.3 km) and were from hosts of both sexes, different ages, and relatedness. The metadata associated with individuals in the main study grid Kloo (KL, *n* = 549 samples) are listed in Table 1. After rarefaction to 4000 reads/sample, we sorted high-quality reads into 12,833 operational taxonomic units (OTUs) using a sequence identity cutoff of 97%. On average, we detected 575 ± 139 OTUs per sample.

**Table 1:** Characteristics of red squirrels in main study grid KL (*n =* 549)

Characteristics	Number of samples (individuals)
Sex	
Male	220 (81)
Female	329 (58)
Year	
2008	240 (95)
2009	120 (34)
2010	189 (62)
Season	
Early spring	233 (101)
Late spring	204 (97)
Summer	112 (54)
Age	
0	15 (12)
1	166 (61)
2	110 (38)
3	162 (51)
4	57 (20)
5	37 (8)
6	2 (1)
Pedigree information	
Both dam and sire known	248 (53)
Dam or sire known	116 (20)
Unknown	185 (66)

**Fig. 1:**
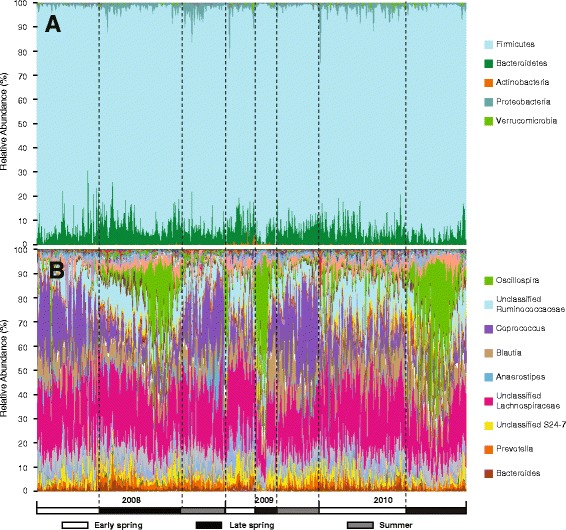
Bacterial composition of 549 red squirrel fecal samples from grid KL (from 2008 to 2010). Each column represents one sample. *Y*-axis values represent the relative abundance of each bacterial taxon. Samples are sorted by the sampling time. **a** Phylum level. **b** Genus level

**Fig. 2:**
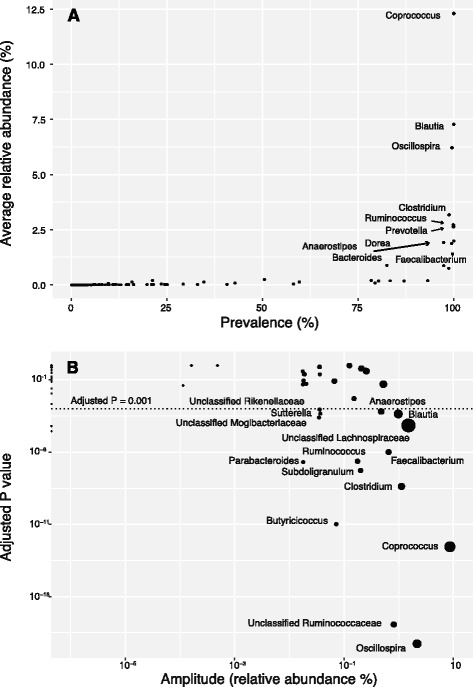
**a** Relative abundance and prevalence of bacterial genera in red squirrel microbiota. The top ten most abundant genera are labeled with their genus names. **b** Bacterial genera showing strong seasonal rhythms. *X*-axis indicates the fluctuation amplitudes, and *Y*-axis indicates the statistical significance of the rhythm. The size of dot represents the average relative abundance of each genus

Taxonomic assignment revealed a fairly typical rodent profile (Fig. 1a): the dominant phyla were Firmicutes (88.6% of total reads), Bacteroidetes (9.0%), and Proteobacteria (1.7%), with 10 rare phyla accounting for the remaining 0.7% of the reads. The prevalence-abundance distribution of genera showed an “L” shape with a heavy long tail toward the left (Fig. 2a), indicating that the most abundant genera were present in almost all samples, while rare genera accounted for most of the membership difference in gut microbiota of red squirrel population. Specifically, the 10 most abundant genera (5% of the total detected genera) were each present in more than 97% of the samples and together made up 41.5% of the total reads: *Coprococcus* (abundance 12.3%, prevalence 100%), *Blautia* (7.3%, 100%), *Oscillospira* (6.2%, 99.6%), *Clostridium* (3.2%, 98.8%), *Ruminococcus* (2.7%, 99.9%), *Prevotella* (2.6%, 99.9%), *Dorea* (2.0%, 100%), *Anaerostipes* (1.92%, 97.4%), *Bacteroides* (1.87%, 99.4%), and *Faecalibacterium* (1.4%, 99.7%) (Fig. 1b). As such, these 10 genera constituted the “core microbiota” of red squirrel gut microbial community. On the other hand, rare taxa appear to be more sample specific. Among the 189 genera detected, 167 (88.4%) were present in less than 50% of samples. Similarly, 11,618 OTUs (90.5% of total OTUs) appeared in less than 10% of samples. On average, only 56% of genera and 20% of OTUs were shared among samples (average Jaccard distance = 0.44 at the genus level, 0.80 at the OTU level).

### Seasonal variation in gut microbiota diversity and composition

We found evidence of seasonal variation in the gut microbiota composition at the genus level that clearly delineated samples collected in early spring (February through April, 314 samples), late spring (May and June, 382 samples), and summer (July and August, 209 samples) (Fig. 1b). Consistently, principal coordinate analysis (PCoA) revealed a clear seasonal pattern in which samples were largely partitioned by season (Additional file 1: Figure S1). PERMANOVA analysis confirmed that season explained significant variation of gut microbial composition (*P* = 0.001, Table 2). The seasonal variation was not simply due to the turnover of the host population because longitudinal data collected from the same individuals also displayed a clear seasonal pattern (within season Jaccard distance 0.75 < between season Jaccard distance 0.79, Wilcoxon rank sum test, *P* < 0.001, Additional file 1: Figure S2).

**Table 2:** PERMANOVA analysis of environmental and host factors in the KL dataset (*n* = 549 samples)

Factors	% variance explained	*P* value
Season	10.0	0.001
Year	5.0	0.001
Sex	0.9	0.001
Age	0.4	0.008

In order to identify key genera that were strongly associated with season, we performed random forest tests. Using genus composition, random forest models were able to differentiate seasons with an overall accuracy of 83% (Table 3). We identified six abundant and three rare genera with strong discriminative power. Interestingly, the top discriminatory genera *Oscillospira*, *Coprococcus*, and *Clostridium* were all core genera.

**Table 3:** Highly discriminative genera for predicting seasons by random forest test

Genera	Mean % increase in error on removal (± SD)	Relative abundance in early spring/late spring/summer
Oscillospira	21.88 (± 1.22)	2.6%/15.4%/1.4%
Butyricicoccus	11.97 (± 1.33)	0.12%/0.29%/0.03%
Coprococcus	11.92 (± 1.04)	13.0%/6.3%/22.8%
Unclassified Ruminococcaceae	10.40 (± 0.81)	10.3%/9.2%/7.5%
Clostridium	10.26 (± 1.22)	1.9%/5.4%/4.0%
Unclassified Coriobacteriaceae	5.1 (± 0.76)	0.015%/0.05%/0.004%
Faecalibacterium	4.89 (± 0.45)	1.1%/2.0%/1.0%
Ruminococcus	4.44 (± 0.54)	0.7%/1.3%/0.6%
Parabacteroides	4.02 (± 0.49)	0.18%/0.05%/0.14%

**Fig. 3:**
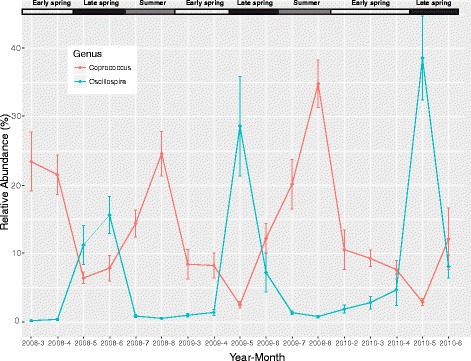
Oscillation of two core genera *Coprococcus* and *Oscillospira* over time. Samples were from the grid KL and binned by month. The error bar represents +/− one standard error of the mean

Using JTK_cycle [43], a non-parametric algorithm for detecting rhythmic elements in circadian clock studies, we confirmed that seasonal fluctuations in the abundance of these key genera were repeatable across years (Fig. 2b). In total, we found 15 genera showing a strong seasonal periodicity of 11–12 months (*P* < 0.001), including all highly discriminatory genera identified in the random forest analysis except unclassified Coriobacteriaceae. Many of the 15 genera were also core genera. Among them, *Coprococcus* and *Oscillospira* exhibited the largest periodic fluctuation in relative abundance (amplitude). As shown in Fig. 3, there was a clear tradeoff of core genera *Oscillospira* and *Coprococcus*, which peaked in late spring and summer, respectively.

The seasonal changes in gut microbiota occurred in parallel with the shift in red squirrel’s dietary composition as measured by opportunistic feeding observations of individually marked squirrels (*n* = 1279 observations between 2008 and 2010; Additional file 1: Figure S3). We estimated the diet composition by aggregating all feeding events within each month and then used the monthly diet composition in the statistical analyses. In early spring, red squirrels mainly consumed seeds from hoarded white spruce (*Picea glauca*) cones and hypogeous fungi (false truffles). In late spring, red squirrels also consumed a significant amount of fresh white spruce buds and needles. In summer, red squirrels began to consume seeds from newly available spruce cones produced in the current year [38]. A Mantel test showed that diet and gut microbiota compositions were significantly correlated (Bray-Curtis distance, *r* = 0.44, *P* = 0.003). To further explore what specific components of the diet correlated with the changes in gut microbial community structure, we analyzed the association of food items with the seasonal rhythmic genera. The elevated level of *Oscillospira* correlated with increased intake of spruce buds in the late spring (*R* ^2^ = 0.36, *P* = 0.007). In contrast, the relative abundance of *Coprococcus* was higher at times of year when new spruce cones were consumed (*P* = 0.0002), but was negatively associated with spruce buds (*P* = 0.007, total *R* ^2^ = 0.92). The percentage of false truffle mushroom consumption best predicted the levels of *Clostridium* in red squirrel gut microbiota (*R* ^2^ = 0.25, *P* = 0.03) where lower levels of *Clostridium* were associated with greater false truffle consumption.

Alpha diversity (measured by Chao1) also displayed a distinct cyclical pattern (JTK_cycle: adjusted *P* value < 0.0001, period 12 months, amplitude 101.0). Within each year, species richness reached minima in the late spring and maxima in the summer (Additional file 1: Figure S4). Interestingly, the overall microbial species richness decreased from 2008 to 2010, which coincided with the natural decrease in red squirrel population density over these years (2008: 1.46 squirrels/ha; 2009: 1.15 squirrels/ha; 2010: 0.93 squirrels/ha), resulting from several years of low spruce cone production [44].

### Seasonal OTU co-occurrence network

To investigate how species interactions and the structure of red squirrel gut microbial community changed over time, we reconstructed OTU co-occurrence network in each season. Analyses of OTU network revealed scale-free network structures in all three seasons (power law, *R* ^2^ > 0.6). Despite the overall similarity in network structure (Additional file 1: Table S1), the key hub species (species with most connections to other species) varied from season to season, indicating distinct species-species interactions in each season (Additional file 1: Figure S5). In early spring, a *Coprococcus* species (OTU 21475) was the most dominant hub in the network. In late spring, it faded out of the network and an *Oscillospira* species (OTU 54301) became the most dominant hub. Nevertheless, there was still notable continuity in network structures. For example, early spring and late spring both had OTU 47644 (unclassified Ruminococcaceae) as a prominent hub. Late spring and summer had more hubs in common: OTU 67162 (*Dorea*), OTU 100783 (*Clostridium*), and OTU 32425 (unclassified Ruminococcaceae). Summer and early spring both had OTU 21475 as their most dominant hub. Interestingly but not surprisingly, most of the hub species belonged to the core genera.

### Effect of food supplement on red squirrel gut microbiota

In three out of the six study grids, peanut butter was supplied from October to May in a large-scale food supplementation experiment [44]. In our 3 years (2008, 2009, 2010) of sampling feces from squirrels on the three control (no food added) and three experimental (peanut butter added) grids, the average population density in the grids with food supplement was twofold higher compared to the other three grids without supplement (with supplement, 3.13 squirrels/ha; without, 1.58 squirrels/ha). To assess the effects of food supplement on gut microbiota, we performed PCoA analysis of 225 samples collected in May 2008 from female squirrels in all six grids. Samples displayed clear separation by food supplement group (Additional file 1: Figure S6). Grids with food supplement had significantly more genera *Sutterlla* and *Ruminococcus*, and less *Coprobacillus*, *Clostridium*, and *Anaerostipes* compared to samples collected from squirrels on the control grids with no supplemental food (Wilcoxon rank sum test, FDR adjusted *P* < 0.05). Food supplemented squirrels also had significantly lower alpha diversity (Chao1) than unsupplemented control squirrels (food supplement 982.9 ± 263.7, control 1094.4 ± 301.4, non-parametric *t* test, *P* = 0.018).

### Biogeography of red squirrel gut microbiota

To investigate whether red squirrel gut microbiota had any spatial structure, we carried out analyses on the same set of samples (*n* = 225) described above (from females in all six grids that were 0.2–7.3 km apart, May 2008). To control for potential confounding effects of food supplementation and host relatedness (red squirrels disperse ~ 100 m from their natal territory [40]), we only compared samples within the same treatment group (from a food-supplemented or control grid) and from unrelated individuals (relatedness coefficient *r*~0). Only one sample from each red squirrel was included.

PERMANOVA analysis performed on grids within the same food group confirmed that grid was a significant predictor of microbiota beta diversity (data not shown). Consistently, between-grid distances were significantly higher than within-grid distances (Jaccard distance: within-grid 0.75, between-grid 0.77, *P* < 0.0001, Wilcoxon rank sum test).

**Fig. 4:**
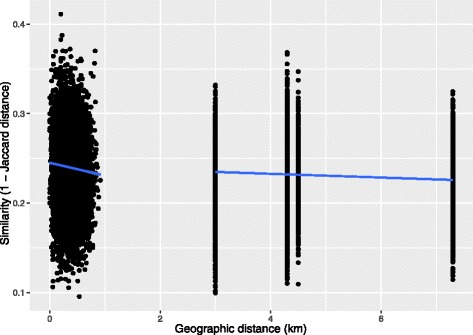
Distance-decay of the red squirrel gut microbial communities within and between grids. Each dot represents a comparison between samples collected at different geographic locations. *Y*-axis represents the microbiota similarity. The lines denote the linear regressions of microbial similarity over the geographic distance

Next, we investigated the effect of geographic distance on microbial diversity. Linear regression analysis revealed significant similarity-distance decays for both within and across grids (Fig. 4). Every 1 km increment in geographic distance resulted in 1.4% increase in Jaccard distance within grids (*P* < 0.001) and 0.2% increase between the grids (*P* < 0.001).

### Individuality and maternal effects on gut microbiota

Previous studies have shown that family members tend to have more similar gut microbiota than unrelated individuals and increased levels of host relatedness are associated with greater similarity in gut microbial communities [6, 9, 27, 28]. We tested the effect of kinship and genetic relatedness on microbial diversity, taking advantage of the comprehensive pedigree information available for our red squirrel study population [45]. To eliminate any seasonal or spatial effects, all comparisons were performed between samples from the same study grid (KL) within the same year and season. We analyzed a total of 121 self-pairs (relatedness coefficient *r* = 1) sampled at different times (average 19.8 days apart), 59 mother–offspring pairs (*r* = 0.5), 35 father-offspring pairs (*r* = 0.5), 13 full sibling pairs (*r* = 0.5), 77 pairs of half-siblings (*r* = 0.25, maternal 37, paternal 40) and 1293 pairs of unrelated individuals (*r*~0).

**Fig. 5:**
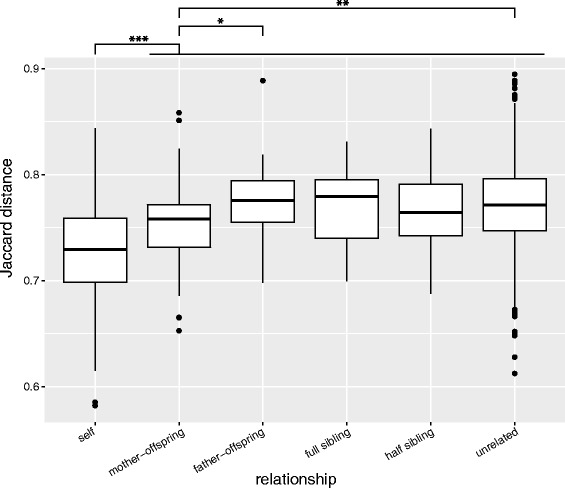
Red squirrel gut microbiota exhibit individuality and maternal effects. Box-and-whisker plots show pairwise Jaccard distances within each relationship groups. Significance values are from non-parametric Kruskal-Wallis tests (FDR adjusted). *P < 0.05, **P < 0.01, ***P < 0.001

We found evidence of individual gut microbiota signatures. We analyzed multiple samples (median 12, range 9–26 samples per individual) from the same squirrels (*n* = 21) collected over a time span ranging from 83 days to 828 days. Samples collected from the same individual at different time points were more similar to each other than to other individuals (Fig. 5, Wilcoxon rank sum test, FDR adjusted *P* = < 0.0001).

Interestingly, except for the mother-offspring pair, microbial similarities of all other related pairs (i.e., father-offspring, half siblings, and full siblings) were not significantly different from unrelated pairs (mother-offspring, FDR adjusted *P* = 0.01; other related pairs, FDR adjusted *P* > 0.1, Fig. 5), indicating that genetic relatedness of the host did not affect the gut microbial composition. Consistently, Mantel test did not find any significant correlation between host relatedness and microbial similarity. On the other hand, mother-offspring pairs had significantly higher microbial similarity compared to all other pairs including the father-offspring pairs (Wilcoxon rank sum test, FDR adjusted *P* = 0.03) (Fig. 5).

### Relative contribution of environmental and host factors

We next performed PERMANOVA on Bray-Curtis beta diversity to assess the relative contribution of environmental factors (year and season) and host factors (sex and age). Our results revealed that overall the environmental factors explained ~ 11 times more variation in gut microbial community structure than the host factors (Table 2). As expected, season had the most explanatory power, explaining around 10.0% of the total variation. Year was next, accounting for 5.0% of variation. Although statistically significant, contributions of host sex and age were small, with each explaining no more than 0.9% of variation.

## Discussion

In this study, we focused on a well-characterized red squirrel population to assess the contribution of environmental and host factors in shaping gut microbiota structure. At the phylum level, red squirrel gut microbiota composition is broadly similar to those of other mammalian gut microbiota [13, 14, 46], with Firmicutes (88.6%) and Bacteroidetes (9.0%) being the two major phyla. This result is consistent with our present understanding that the mammalian gut harbors a highly constrained set of bacterial phyla adapted to the gastrointestinal tract condition [13]. Unlike wild wood mice (*Apodemus sylvaticus*), which are dominated by the genus *Lactobacillus* [15], red squirrels have a high level of the genera *Coprococcus* (12.3%) and *Oscillospira* (6.2%), but very low levels of *Lactobacillus* (0.88%). Notably, red squirrels have remarkably low variation in gut bacterial phyla and shared a core set of genera across time (year, season, host age), space (study grid), and host family (Fig. 2a).

We found a remarkable seasonal rhythm in red squirrel gut microbial composition, manifested by a tradeoff of the relative abundance of two core genera, *Oscillospira* and *Coprococcus* in late spring and summer (Fig. 3). Consistently, the network analyses revealed shifts in key hubs in late spring from *Coprococcus* to *Oscillospira* and a swap in summer (Additional file 1: Figure S5). The seasonal rhythm in gut microbial structure is clearly associated with seasonal dietary changes. The shift in microbiota composition coincides with emergence of fresh spruce buds in late spring and fresh spruce cones in summer (Additional file 1: Figure S3). Accordingly, we found that the level of *Oscillospira* was positively correlated with consumption of fresh spruce buds, whereas the level of *Coprococcus* was positively correlated with consumption of fresh spruce cones and negatively correlated with consumption of fresh spruce buds. *Oscillospira* are frequently found in cattle and sheep rumen and increase significantly in relative abundance when hosts are feeding on fresh forage diet [47]. This is consistent with our findings and strongly suggests that fluctuation of *Oscillospira* is driven by diet. Our result corroborates the findings in the wild wood mice and a human hunter-gathers population [15, 48]. Both studies observed seasonal cycling in the gut microbiota composition, which correlated with the seasonal shift in food availability and diet.

The fact that *Oscillospira* and *Coprococcus* are present in virtually all the samples we surveyed suggests that they were long-term gut residents and not foodborne. Supporting this, there was no evidence of *Oscillospira* on fresh forage (pasture grass) fed to cattle or in soil [47]. Our result suggests that red squirrel gut microbiota switch between alternative states in response to recurring seasonal dietary changes. This may have resulted from continuous selective pressure on gut microbial community during host-microbiota coevolution. Gut microbiota adapted to seasonal dietary shift can rapidly shift their metabolic activity, provide the host dietary flexibility, maximize energy extraction, and likely increase the fitness of the host-microbe ecosystem.

Biogeographic patterns have been observed in human and wild mice populations [6, 15, 29, 30]. All biogeographic patterns were detected between populations. We found evidence for a weak but significant spatial structure at a much smaller local scale. Microbial composition varied across six study grids within a few kilometers. Moreover, a similarity-distance decay relationship was found within a population. Distance-decay patterns in microbial communities can be driven by environmental factors that vary across space, as recapitulated by the hypothesis that “everything is everywhere, but the environment selects” [49]. Alternatively, the spatial patterns can be due to dispersal limitation, as it allows historical effects to influence contemporary community structure. We have controlled for potential confounding environmental factors in our analysis (we only included samples from the same year, season and sex). Thus, we think the most likely explanation for this biogeographic pattern is dispersal limitation of gut microbes, although we cannot rule out unmeasured spatially structured environmental factors. Red squirrels defend exclusive territories year-round and have relatively limited physical interactions except during mating [39, 50]. Thus, it is not surprising that red squirrel gut microbiota might be constrained by stronger dispersal limitation, which could result in spatial structures at a small local scale.

Island biogeography theory [51] can be useful for understanding the microbial diversity if we view each individual gut as an island. Island theory posits that early colonizers could strongly influence the future community composition. Mothers can make a large contribution to the species pool that first colonizes offspring. It has been recently proposed that maternal transmission of gut microbiota is universal in animals [52] and the effects of maternal transmission can be manifested over several generations [34]. In our study, we found that gut microbiota of red squirrels were significantly more similar to those of their mother than to those of their father (males provide no paternal care) and unrelated individuals. This finding indicates not only that gut microbiota in red squirrel can be maternally transmitted but also that the maternal effect persists until adulthood. Our result differs from a recent study showing no maternal effect in a chimpanzee population [26]. This can be explained by infrequent physical interactions between red squirrels compared to the highly social chimpanzees. It has been suggested that over the course of a lifetime, chimpanzees acquire most of their gut microbiomes through social interactions [26]. Also in contrast to findings in human populations [9], we found no evidence indicating host genetics influence gut microbiota diversity in red squirrels. Since mother and father were equally related to offspring, the genetic relatedness cannot explain higher microbial similarity observed in mother-offspring pairs. In addition, gut microbiota were not significantly different between father-offspring, full sibling, half sibling, and unrelated individual pairs.

## Conclusions

In summary, we performed a comprehensive survey of gut microbiota of a well-studied wild red squirrel population. Red squirrels harbor a typical rodent gut microbiota with a stable set of core genera. However, these core genera exhibited a remarkably strong seasonal rhythm in their relative abundance mainly associated with seasonal dietary changes that was consistent across 3 years of study. We also found significant biogeographic pattern in gut microbial structure at a fine local scale (meters to kilometers) indicative of limited gut microbial dispersal. Despite the dominant effect of environmental factors, we found clear signatures of individuality and maternal effects in red squirrel gut microbial communities. However, host genetics does not seem to be a significant driver. Taken together, the results of this study emphasize the importance of external ecological factors rather than host attributes in driving temporal and spatial patterns of gut microbiota in natural environment.

## Methods

### Sample collection

#### Subject description

Study subjects were from a natural population of North American red squirrels in the southwest Yukon (61°N, 138°W) near Kluane National Park. Red squirrels in this area have been continuously monitored by the Kluane Red Squirrel Project since 1987 using a combination of live-trapping and behavioral observations. All squirrels were permanently marked with small metal ear tags and regularly monitored from March to September of each year. Several types of data including identity, sex, body mass, reproductive status, territory ownership, and dietary information were collected [37, 38]. In this study, we collected 1000 fecal samples from 363 individuals that spanned 3 years and 240 ha. Samples used in our study were described below. A detailed description of the population can be found in [37].

*Study grids.* The study area consists of six ~ 40 ha grids that are 0.2~7.3 km apart from each other (Additional file 1: Figure S7). Samples were collected mainly from two grids (Kloo or KL, *n* = 618; Agnes or AG, *n* = 232). Samples collected from the other four grids were used to study the biogeographic structure of microbiota between grids (Jo or JO, *n* = 25, Sulfur or SU = 25, Chitty or CH = 50, Lloyd or LL = 50). On grids AG, LL, and JO, peanut butter was provided as a food supplement from October to May in each year to experimentally increase population density [44]. The three other study grids (KL, CH, SU) were not manipulated and, therefore, represented the natural environment for red squirrels. All study grids were staked and flagged at 30 m intervals so that we could identify the exact distances among the territories of different squirrels.

#### Sampling years and seasons

The seeds of white spruce cones (*Picea glauca*) are the major food resource for red squirrels [38]. White spruce is a masting tree species that produces a super-abundance of cones in some years (mast years) and few to no cones in other years [53]. Previous studies have shown tremendous yearly variation in spruce cone production that has large ecological and evolutionary impacts on red squirrel life histories and behavior [38, 44, 54]. Our samples span from year 2008 to 2010, with 2010 being a mast year when spruce cone production was extremely high, though new cones produced in that year were largely unavailable until mid-August. Within each year, samples were collected in three seasons: early spring (February through April), late spring (May and June), and summer (July and August).

#### Diet information

The study area is located in the Boreal forest, dominated by white spruce and willows (*Salix spp*.). Red squirrels mostly feed on the seeds of white spruce cones. White spruce trees produce fresh cones that are available to be consumed by red squirrels from July to September of each year. These fresh spruce cones are hoarded for subsequent consumption in a larder hoard at the center of the squirrel’s territory (midden). Squirrels consume these cached cones from their midden throughout the winter and at least into the next spring. Squirrels also feed on mushrooms, spruce buds, false truffles, berries, and a variety of items depending on their seasonal availability [38]. From March–September of each year, we opportunistically recorded what food items squirrels were feeding upon and visually identified these items. We classified these items of red squirrels’ diet into five food categories: (1) seeds of hoarded white spruce cones, (2) seeds of fresh white spruce cones, (3) hypogeous fungi (false truffles), (4) spruce buds, and (5) others (spruce needles, spruce bark, willow leaves, willow buds, aspen leaves, bearberry flowers, white spruce witches broom rust caused by the fungus *Chrysomyxa arctostaphyli*, animal material, snow, and unidentifiable items) (Additional file 1: Figure S3). In total, we recorded 1279 feeding events spanning 16 months between 2008 and 2010 (average 80 events per month). Since diet composition is similar among individuals, all feeding events were aggregated by months in our study to provide suitable estimates of the composition of diet for each month.

#### Territory

Both female and male adult red squirrels defend exclusive territories around a central larder hoard (midden) containing cached white spruce cones for over winter survival [39]. Juveniles that fail to acquire territories before their first winter generally do not survive [55]. On average, only 26% of offspring survive to 1 year of age [37, 56]. In May and August of each year, we completely enumerated all squirrels on the study grids so that we could identify territory ownership. The location of each animal’s midden was recorded and used to estimate the distance between individuals.

#### Age

The average adult lifespan of wild red squirrels in this population is 3.5 years (maximal, 9 years) [37]. Juveniles usually leave the natal area 70 days after birth, and the mean dispersal distance is 96 ± 94 m from the natal area [40]. Red squirrels reach sexual and reproductive maturity at ~ 1 year old. After adults acquire a territory prior to experiencing their first winter, dispersal away from that territory is rare [40]. In this study, we collected samples from individuals 0–6 years of age. The age estimation for most individuals was accurate to within days because most individuals were first marked in their natal nest.

#### Sex and reproductive status

Red squirrels are sexually monomorphic in adult body mass, and there is no sex-bias in natal dispersal [57]. During the breeding season (roughly March to May depending upon the year), multiple males come to the territories of females to mate with them [58]. Females raise young without any help from males. During each capture event, we recorded the reproductive status of adult females through abdominal palpation (to assess pregnancy status as fetuses are palpable through the abdomen) and assessing nipple status. Females were categorized as non-breeding, lactating, or weaning.

#### Pedigree

For the fecal samples that we collected between 2008 and 2010, the multigenerational pedigree [45] for the KL grid included 124 individuals, with 78 known maternal links and 83 known paternal links. Maternity was determined by behavioral observation before the emergence of juvenile squirrels from their natal areas. Paternity was assigned based on 16 microsatellite loci using CERVUS 3.0 with > = 95% confidence (detailed in [58]).

### Fecal sample collection, DNA extraction, and 16S rRNA sequencing

Fecal samples collected from underneath live traps were placed into 1.5 mL vials individually using forceps. Fecal samples collected in the colder months (January–April) were generally frozen upon collection. In the warmer months (May–September), the vials were kept on ice and then transferred to a − 20 °C freezer within 5 h of collection [59].

We extracted DNA from fecal samples in a 96-well format using the ZR-96 Fecal DNA Kits (Zymo Research, Orange, CA) following the manufacturer’s protocol. The V1–V3 hypervariable regions of the 16S rRNA gene were amplified using two universal primers 27F (5’-AGRGTTTGATCMTGGCTCAG-3′) and 534R (5’-TTACCGCGGCTGCTGGCAC-3′). We added a unique 8 bp barcode to each primer to tag the samples and used a 50-uL reaction for each PCR amplification by QIAGEN Taq polymerase (Qiagen Inc., CA). PCR conditions consisted of 94 °C for 3 min, followed by 25 cycles of 94 °C for 30 s, 57 °C for 30 s, and 72 °C for 60 s, with a final extension of 5 min at 72 °C. 16S rRNA amplicons from different samples were pooled in equal molar ratios, then gel purified and sequenced on an Illumina MiSeq platform using the 300 bp paired-end (PE) protocol. All liquid transfer steps were performed on a Biomek NXp liquid handling station (Beckman-Coulter Inc., Fullerton, USA).

### Sequence processing, quality control, and OTU classification

We filtered sequence reads by base quality using TRIMMOMATIC 0.32 with settings of LEADING = 3, SLIDINGWINDOW = 10:20, and MINLEN = 50 [60]. Paired-end reads passing the quality filter were merged using FLASH (-r 301 -f 447 -s 45 -x 0.05) [61]. The successfully merged reads were assigned to samples by barcodes and processed using the QIIME pipeline [62]. We identified chimeric sequences using USEARCH [63] implemented in QIIME with both de novo and reference-based detection algorithms. Only those sequences that were flagged as non-chimeras with both detection methods were retained. We then removed non-16S rRNA sequences using hmmsearch [64] against a custom-made 16S rRNA gene model. The remaining reads were clustered to OTUs by UCLUST [65] using an identity threshold of 97%. The most abundant sequence of each OTU was selected as the representative sequence, which was then classified using the RDP classifier [66]. OTUs belonging to mitochondrion or chloroplast were removed. To remove sequencing effort heterogeneity, samples were rarefied to 4000 reads per sample. Of our initial set of 1000 samples, three were excluded as outliers because the average distance of each of these three samples from other samples were more than 1.5 times the interquartile range above the higher 75% percentile. During rarefaction, 92 samples were removed due to insufficient number of reads, leaving a final dataset of 905 samples.

### Effect of environmental/host factors on gut microbial diversity

We measured alpha diversity by Chao1 index. To compare beta diversity among samples, we first excluded any OTUs with less than five sequence reads. We then constructed beta diversity matrices from OTU table using four distance metrics: Jaccard, Bray-Curtis, unweighted UniFrac and weighted UniFrac distance [67].

To test the predictors of gut microbial composition, we first performed exploratory PCoA using all beta diversity matrices in QIIME. To assess the relative contribution of environmental and host factors to the variation of microbial community, we performed PERMANOVA on Bray-Curtis distance matrices of 549 samples in the KL grid using the “adonis” function of the vegan package implemented in R [68]. Factors included in the PERMANOVA analysis included season, year, host age, and sex. The percentage of variation explained by each factor was measured using *R* ^2^, and the significance (*P* value) of each factor was obtained by 999 permutation tests.

### Identifying bacterial taxa with seasonal rhythms

We used supervised random forest model implemented in QIIME (supervised_learning.py) to identify signature genera in each of the three seasons: early spring, late spring, and summer. Random forest model classified each fecal sample into one of three seasons using models built on the relative abundance of each genus. Model accuracy was calculated using the 10-fold cross validation error estimate, which was an approximation of how frequently a sample was misclassified. The discriminatory power of each genus was assessed by comparing the classification accuracy with and without including the genus in the model. Genera that led to more loss of classification accuracy were considered to be more discriminatory.

To test whether bacterial genera identified above had seasonal rhythms, we used a non-parametric test JTK_CYCLE [43]. JTK_CYCLE has been used in detecting rhythmic elements in circadian clock studies [69]. We tested seasonal periodicity using a window of 11–12 months. Benjamini-Hochberg procedure was used to control the false discovery rate.

### Correlation between microbial composition and diet

To test possible associations between dietary items and rhythmic genera identified above, we constructed general linear models on each genus. We began with the full model including all dietary items as the explanatory variables and genus relative abundance as the response variable. Non-significant predictor variables were excluded stepwise from the saturated model using the ‘step’ command, and the best model with the lowest AIC score was selected. We checked model assumptions by examining the distribution of residuals and plotting fitted values against residuals. We also performed Mantel tests to evaluate the correlation between the distance matrices built based on dietary item variation and bacterial beta diversity distances. The significance of Mantel’s *r* was assessed with 999 permutations.

### Effect of kinship on gut microbiota

To assess the effects of genetic relatedness, we calculated pairwise relatedness from the extensive pedigree data available for red squirrels in the KL grid [45] using the R package pedantics [70]. We then performed Mantel tests to evaluate the correlation between the relatedness matrix and microbial beta diversity distance matrices. To further assess the effect of kinship, we divided pairs into six groups: self-pairs (relatedness coefficient *r* = 1, *n* = 121), mother-offspring (*r* = 0.5, *n* = 59), father-offspring (*r* = 0.5, *n* = 35), full siblings (*r* = 0.5, *n* = 13), half siblings (*r* = 0.25, *n* = 77), and unrelated (*r*~0, *n* = 1293). Jaccard distances of each group were compared with non-parametric Kruskal-Wallis tests with post hoc comparisons and corrected using Benjamini-Hochberg false discovery rate (FDR). To control for temporal variation, we restricted all the above analyses to only comparing samples collected within the same year and season.

### OTU co-occurrence network

Microbial network of significant co-occurrence and co-exclusion interactions was built using the CoNet 1.1.0 plugin [71] in Cytoscape [72]. Networks were built for each season separately, and only abundant OTUs (average relative abundance > 0.1%) were used. The analyses were carried out with the following parameters: 1000 initial top and bottom edges, five similarity measures (Spearman, Pearson, Mutual information; Kullbackleibler, and Bray Curtis), null distribution generated by 1000 permutations with renormalization, and 1000 iterations for bootstraps. Networks built with different similarity measures were merged using the Simes method [73] and a Benjamini-Hochberg false discovery rate (FDR) cutoff of 0.05. NetworkAnalyzer was used to analyze the topological parameters of the resulting networks [74].

## Additional file

Additional file 1:
**Figure S1.** Principal coordinate analysis (PCoA) of red squirrel gut microbial communities in grid KL based on Bray-Curtis distance. Samples are colored by sampling season. The percentage of the variation explained by the first three coordinates is indicated on the axes. **Figure S2.** Time-decay of the red squirrel gut microbial communities. Each dot represents a comparison between two samples of the same individual collected at different time points. The colors of dots represent the combination of seasons when the two samples were collected. *Y*-axis represents the microbiota similarity. The similarity decay as a function of time best fits a power law (blue line). The shade shows the 95% confidence bounds. **Figure S3.** The composition of red squirrel diet across 3 years aggregated by month. Each color represents a different dietary item. **Figure S4.** Seasonal rhythm in the alpha diversity of red squirrel gut microbiota. Species richness is estimated by Chao1 index. **Figure S5.** Key hub species in OTU co-occurrence network vary by season. The co-occurrence network is displayed using Cytoscape with the Prefuse Force Directed (edge betweenness) layout. Negative correlations are represented by red edges and positive correlations by green. Each node represents an OTU with > 0.1% relative abundance and is colored by bacterial family to which it belongs. Key hub OTUs are labeled with their IDs, genus names, and the numbers of positive and negative edges. **Figure S6.** Principal coordinate analysis (PCoA) of red squirrel gut microbial communities across six grids based on Jaccard distance. Samples are colored by food supplement status. The percentage of the variation explained by the first three coordinates are indicated on the axes. **Figure S7.** Locations of study grids along the Alaska Highway in the southwest Yukon (61°N, 138°W) near Kluane National Park. (Adopted from 73) Each grid is labeled with the number of samples collected and the food supplement status. **Table S1.** Properties of OTU networks in three seasons. (PDF 903 kb)

## Abbreviations

OTU: Operational taxonomic unit
PCR: Polymerase chain reaction
