# Supplementary material for: Seasonal, spatial, and maternal effects on gut microbiome in wild red squirrels
Source: Microbiome. 2017 Dec 21;5:163. doi: 10.1186/s40168-017-0382-3 (PMC5740981; doi:10.1186/s40168-017-0382-3)
Supplement: Additional file 1: — Figure S1. Principal coordinate analysis (PCoA) of red squirrel gut microbial communities in grid KL based on Bray-Curtis distance. Samples are colored by sampling season. The percentage of the variation explained by the first three coordinates is indicated on the axes. Figure S2. Time-decay of the red squirrel gut microbial communities. Each dot represents a comparison between two samples of the same individual collected at different time points. The colors of dots represent the combination of seasons when the two samples were collected. Y-axis represents the microbiota similarity. The similarity decay as a function of time best fits a power law (blue line). The shade shows the 95% confidence bounds. Figure S3. The composition of red squirrel diet across 3 years aggregated by month. Each color represents a different dietary item. Figure S4. Seasonal rhythm in the alpha diversity of red squirrel gut microbiota. Species richness is estimated by Chao1 index. Figure S5. Key hub species in OTU co-occurrence network vary by season. The co-occurrence network is displayed using Cytoscape with the Prefuse Force Directed (edge betweenness) layout. Negative correlations are represented by red edges and positive correlations by green. Each node represents an OTU with > 0.1% relative abundance and is colored by bacterial family to which it belongs. Key hub OTUs are labeled with their IDs, genus names, and the numbers of positive and negative edges. Figure S6. Principal coordinate analysis (PCoA) of red squirrel gut microbial communities across six grids based on Jaccard distance. Samples are colored by food supplement status. The percentage of the variation explained by the first three coordinates are indicated on the axes. Figure S7. Locations of study grids along the Alaska Highway in the southwest Yukon (61°N, 138°W) near Kluane National Park. (Adopted from 73) Each grid is labeled with the number of samples collected and the food supplement status. Table S1. Properties of O [file 40168_2017_382_MOESM1_ESM.pdf]

Supplementary information

Figure S1. Principal coordinate analyses (PCoA) of red squirrel gut microbial communities in grid KL based on Bray-Curtis distance. Samples are colored by sampling season. The percentage of the variation explained by the first three coordinates are indicated on the axes.

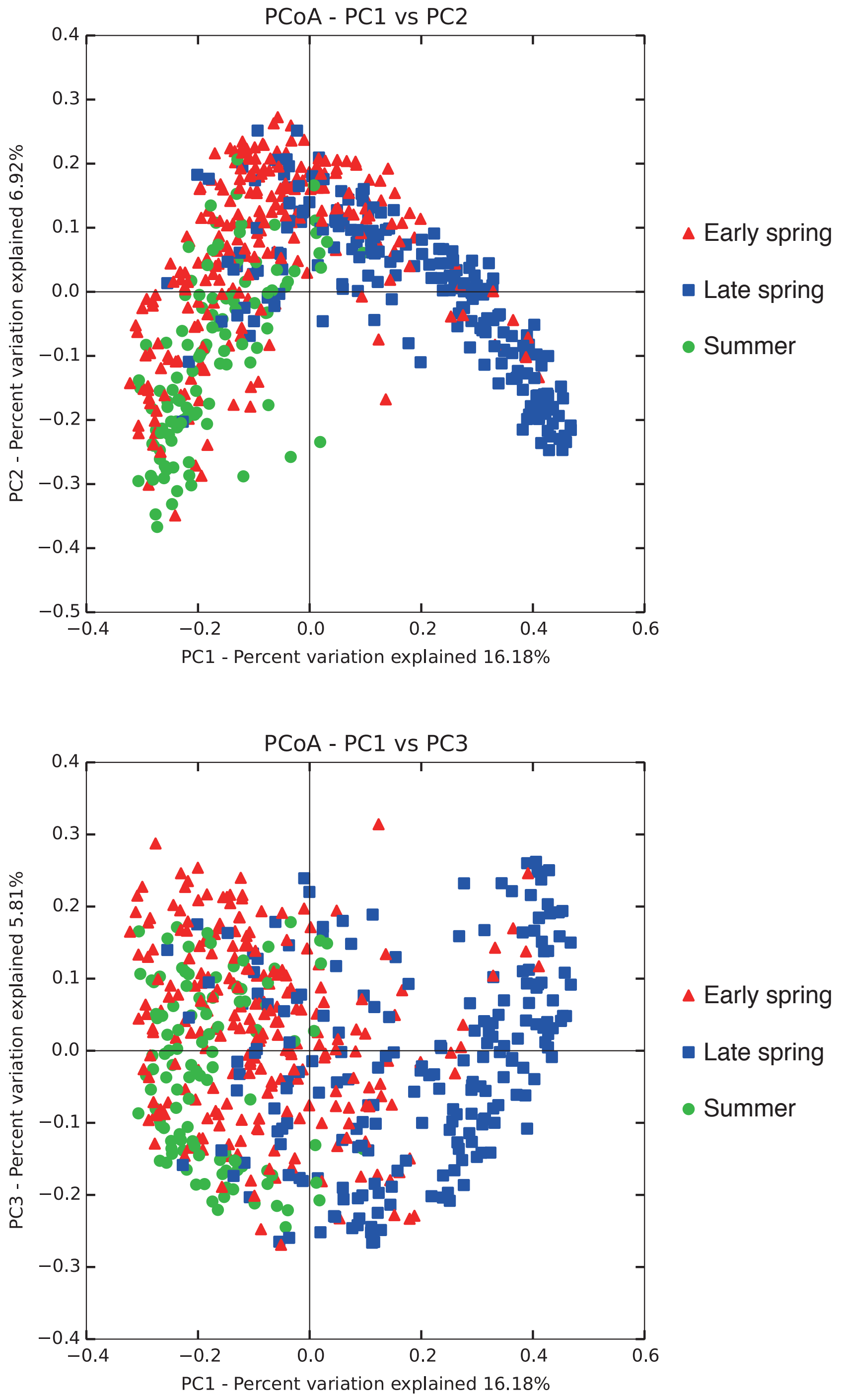

Figure S2. Time-decay of the red squirrel gut microbial communities. Each dot represents a comparison between two samples of the same individual collected at different time points. The colors of dots represent the combination of seasons when the two samples were collected. Y-axis represents the microbiota similarity. The similarity decay as a function of time best fits a power law (blue line). The shade shows the 95% confidence bounds.

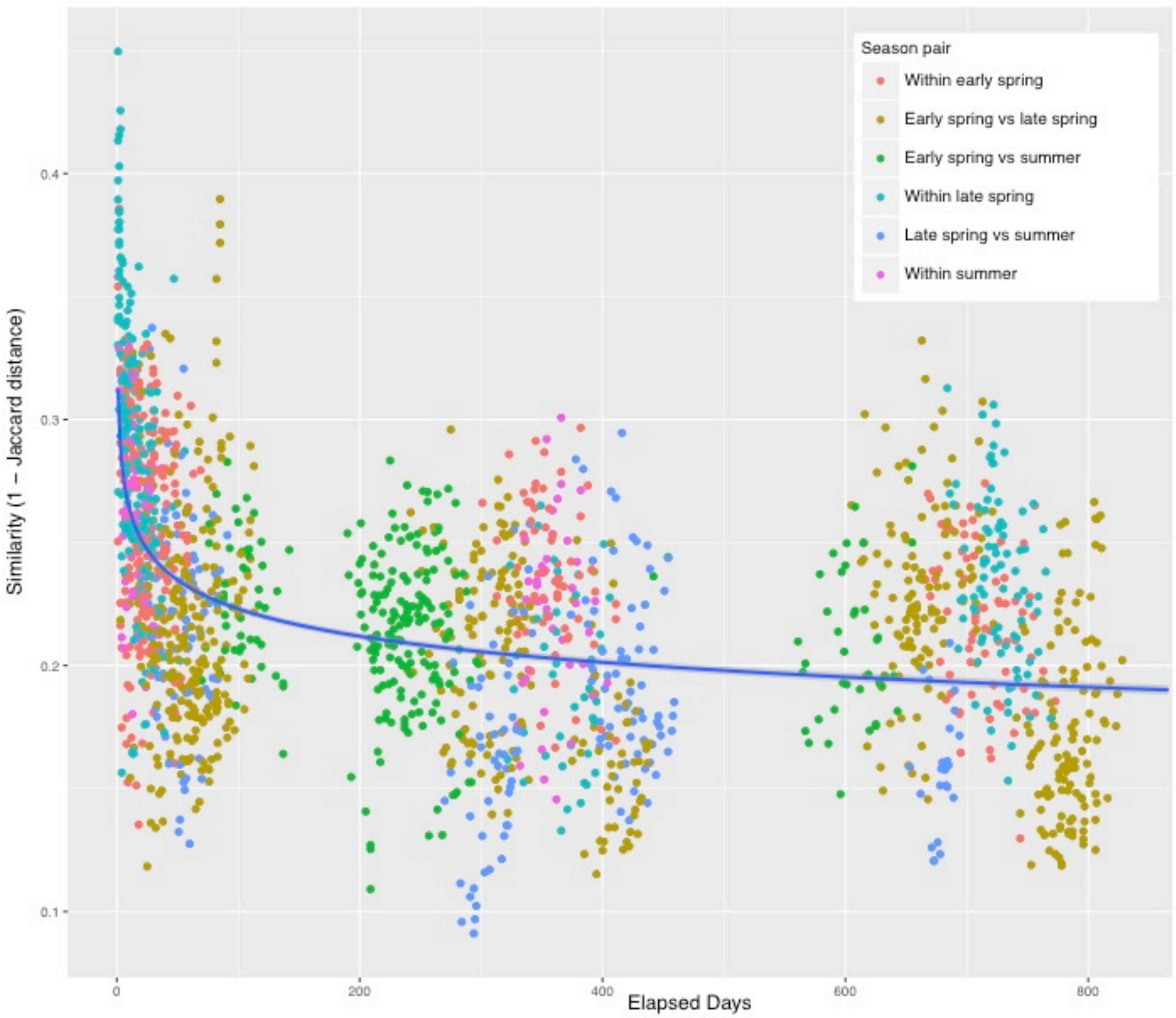

Figure S3 The composition of red squirrel diet across three years aggregated by month. Each color represents a different dietary item.

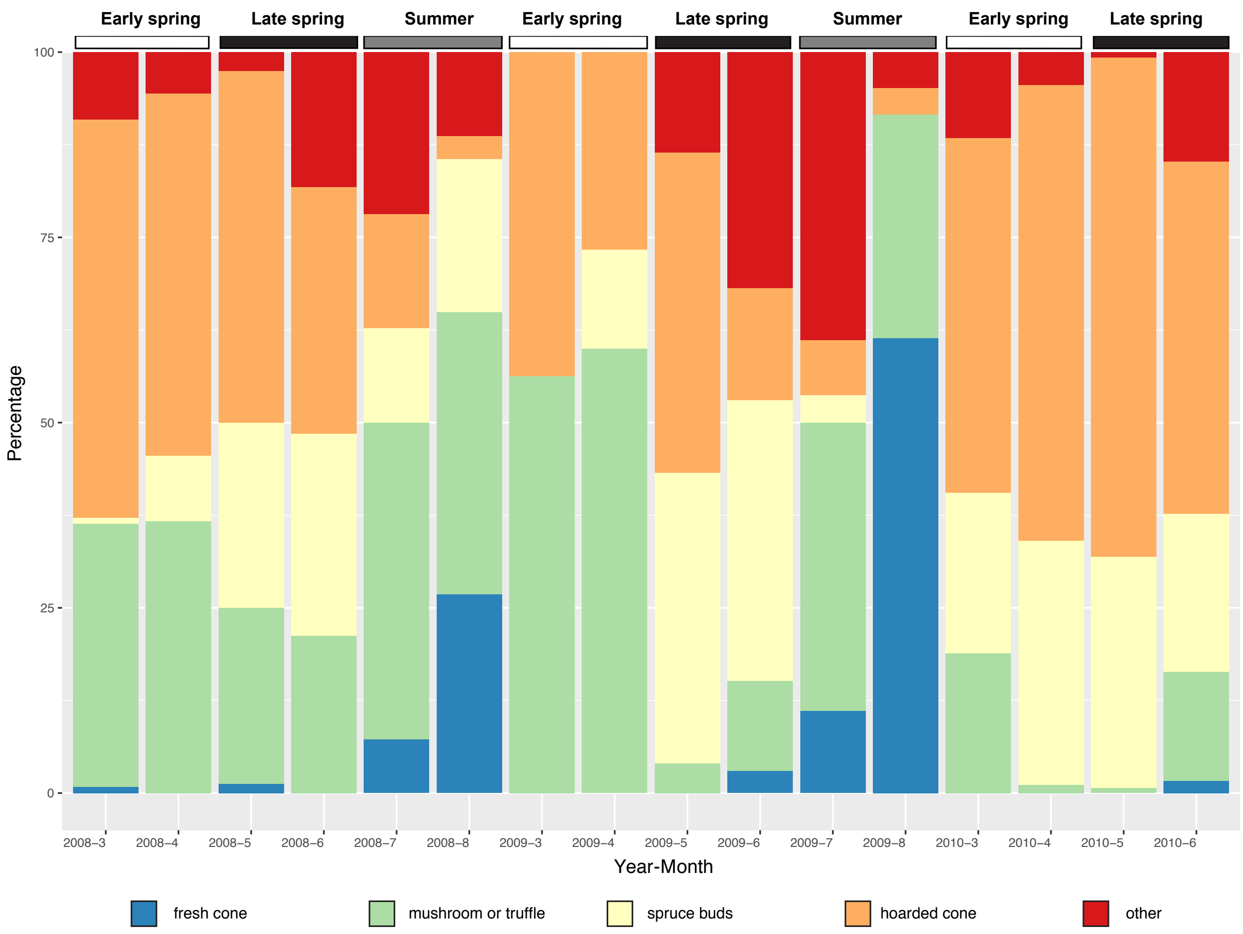

Figure S4. Seasonal rhythm in the alpha diversity of red squirrel gut microbiota. Species richness is estimated by Chao1 index.

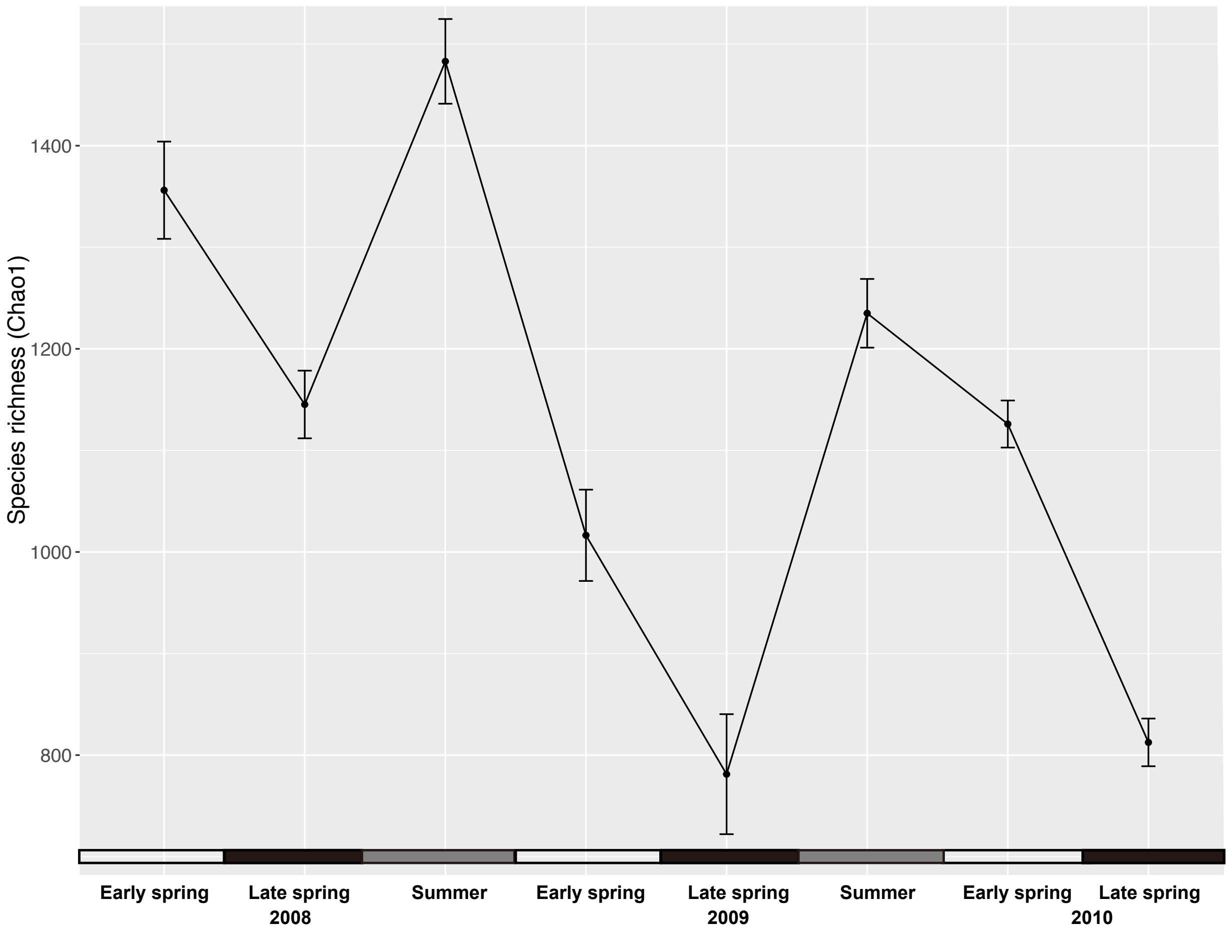

Figure S5. Key hub species in OTU co-occurrence network vary by season. The co-occurrence network is displayed using Cytoscape with the Prefuse Force Directed (edge betweenness) layout. Negative correlations are represented by red edges and positive correlations by green. Each node represents an OTU with >0.1% relative abundance and is colored by bacterial family to which it belongs. Key hub OTUs are labeled with their IDs, genus names and the numbers of positive and negative edges.

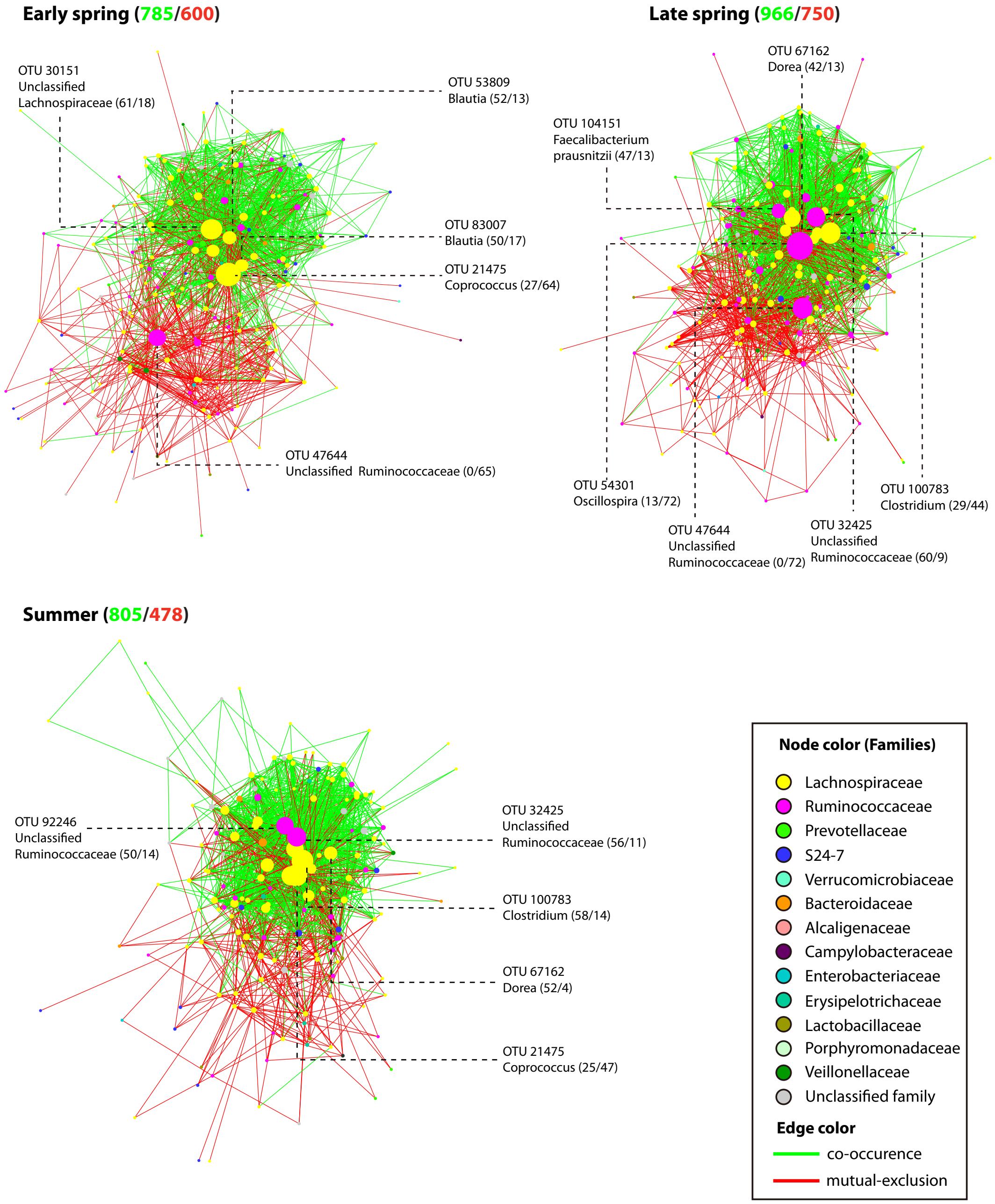

Figure S6. Principal coordinate analyses (PCoA) of red squirrel gut microbial communities across 6 grids based on Jaccard distance. Samples are colored by food supplement status. The percentage of the variation explained by the first three coordinates are indicated on the axes.

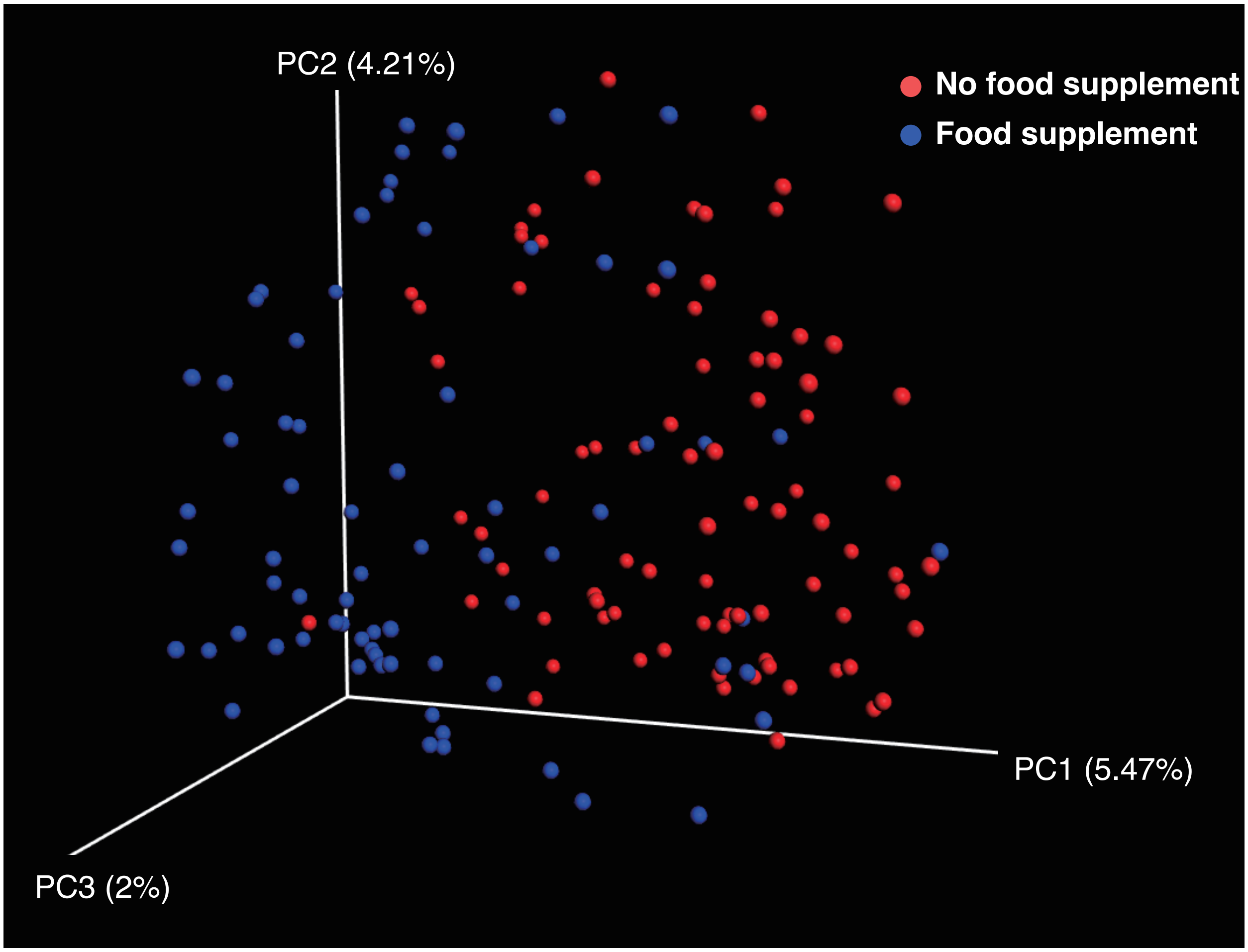

Figure S7. Locations of study grids along the Alaska Highway in the southwest Yukon (61° N 138° W) near Kluane National Park. (Adopted from [73]) Each grid is labeled with the number of samples collected and the food supplement status.

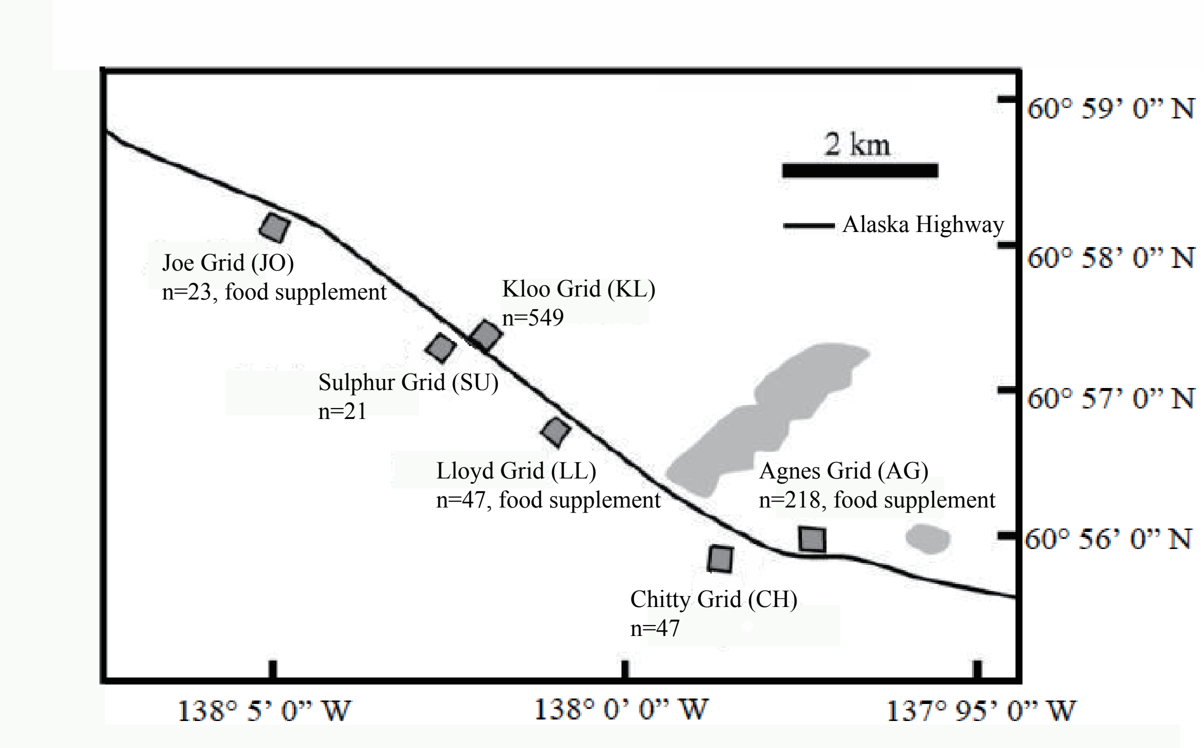

Supplementary Table S1. Properties of OTU networks in three seasons.

Network properties	Early spring	Late spring	Summer
Clustering coefficient	0.354	0.355	0.446
Number of nodes	189	188	162
Number of edges (positive/negative)	1385 (785/600)	1716 (966/750)	1283 (805/478)
Network density	0.078	0.098	0.098
Network heterogeneity	0.967	0.889	0.975
Network diameter	5	4	5
Network radius	3	3	3
Network centralization	0.41	0.361	0.353
Characteristic path length	2.316	2.257	2.297
Average number of neighbors	14.656	18.255	15.84
Shortest paths	35532 (100%)	35156 (100%)	26082 (100%)
